# Complete genome sequence of an Indian outbreak strain of chikungunya virus

**DOI:** 10.1128/mra.00326-24

**Published:** 2024-07-31

**Authors:** Eshna Laha, Deepak Jena, Viplov K. Biswas, Sharad Singh, Sunil K. Raghav, Asit K. Pattnaik, Soma Chattopadhyay

**Affiliations:** 1 Institute of Life Sciences, Bhubaneswar, India; 2 Regional Centre for Biotechnology, Faridabad, India; 3 School of Biotechnology, Kalinga Institute of Industrial Technology (KIIT) University, Bhubaneswar, India; 4 School of Veterinary Medicine and Biomedical Sciences, University of Nebraska-Lincoln, Lincoln, USA; 5 Nebraska Center for Virology, University of Nebraska-Lincoln, Lincoln, USA; DOE Joint Genome Institute, Berkeley, California, USA

**Keywords:** chikungunya virus, CHIKV, whole genome sequence

## Abstract

Here, we report the complete genome sequence of an Indian strain of chikungunya virus isolated from an infected patient from Hyderabad, Andhra Pradesh, India, during a massive outbreak in 2005–2006. The genome length spans 11,811 nucleotides and has a poly(A) tail of 29 residues at the 3′ end.

## ANNOUNCEMENT

Chikungunya virus (CHIKV) (family *Togaviridae*, genus *Alphavirus*) is an arbovirus responsible for debilitating arthritis in humans with no commercially available therapeutics or vaccines (1). India witnessed its first major outbreak in 2005–2006 with nearly 1.3 million people in over 13 Indian states affected to date (2).

A Chikungunya virus Indian outbreak strain (CHIKV-IS) was isolated from a CHIKV-infected patient serum during a rapid outbreak in Hyderabad, Andhra Pradesh, in 2005–2006 and stored at −80°C. The virus was adapted in Vero cells in 2008 through multiple serial passages. Post-adaptation, Vero cells were infected with CHIKV-IS at a multiplicity of infection of 0.1, and plaque assay was performed as described earlier (3). A single well-isolated plaque was picked and resuspended in serum-free media and was used to infect fresh Vero cells. Viral RNA was then isolated from the culture supernatant using the QiaAmp Viral RNA Isolation Kit (Qiagen) as per manufacturer instructions. The sequencing library was prepared using the TruSeq Stranded Total RNA Library Prep Kit (Illumina, USA) following the manufacturer’s protocol. Purified and enriched products were used to create a paired-end next-generation sequencing library and were sequenced using the NovaSeq 6000 platform (Illumina).

Raw BCL files were demultiplexed using the bcl2fastq tool (v2.20.0.422). All tools used for analysis were run with default parameters unless specified. A total of ~12.2 million paired-end reads were generated. The quality of the reads was checked using the FastQC tool (v.0.11.9) (4). The average read length was 36 nt. The adapter sequences and low-quality reads were trimmed and filtered using Cutadapt (v.4.1) for downstream analysis (5). The good quality trimmed reads were mapped to the human genome (GRCh38). All unmapped paired-end reads were extracted using SAMTOOLS (v.1.16.1) and mapped against the CHIKV reference genome (GenBank: DQ443544.2) using BWA mem (v.0.7.17) (6, 7). Deduplication of aligned reads was done using Picard tools (v.2.18.7). Alignment quality was checked using the SAMTOOLS (v.1.16.1) “--flagstat” option. A total of ~2.3 million reads were mapped. We obtained an average coverage depth of >6,000 at each nucleotide position, and the GC content of the final genome was 51%. The nucleotide positions 1,052, 4,167, and 5,049 were confirmed by Sanger sequencing and substituted in the consensus genome file. The consensus genome sequences were derived using the BCFTOOLS (v.1.9) “--consensus” option (8).

**Fig 1:**
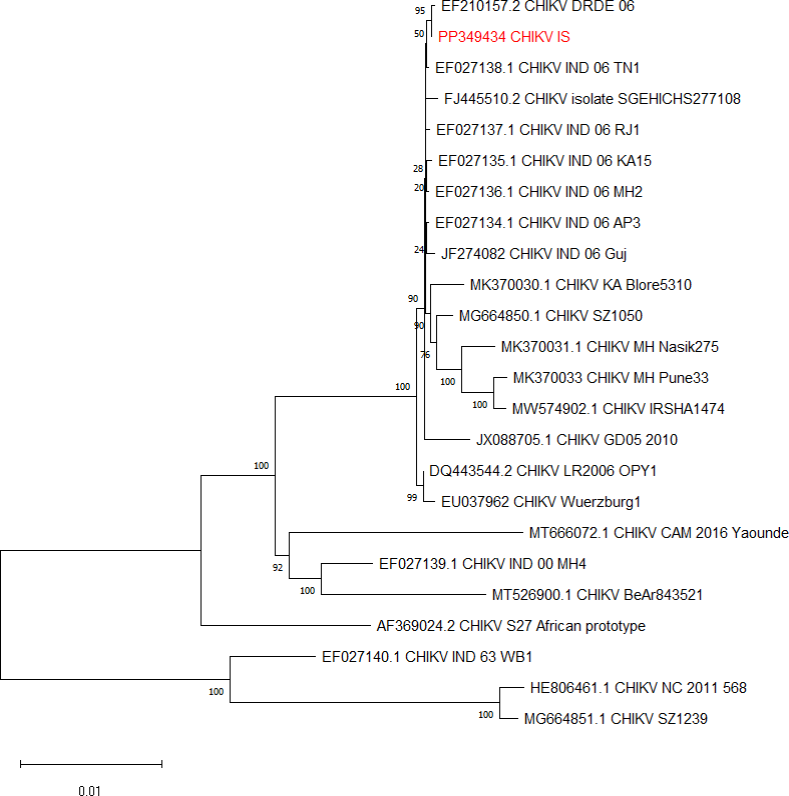
Phylogeny of chikungunya virus strains. Evolutionary history was inferred using the neighbor-joining method with default parameters. The optimal tree is shown and drawn to scale, with branch lengths in the same units as those of the evolutionary distances used to infer the phylogenetic tree. The evolutionary distances were computed using the maximum composite likelihood method and are in units of the number of base substitutions per site. MUSCLE tool was used to build the alignment and cleaned using the “pairwise deletion” option. Neighbor-joining method was the statistical method of choice with 1,000 bootstrap replicates. MEGA11 (v.11.0.13) was used to build and analyze the tree.

The CHIKV-IS genome is 11,811 nt long, followed by 29 adenosine residues at the 3′-terminus. The genome spans non-structural (7,422 nt) and structural (3,744 nt) open-reading frames, separated by a 65-nt-long untranslated region (UTR). The 5′- and 3′-terminal UTRs are 76 and 498 nt, respectively. The CHIKV-IS lacks the signature A226V mutation in the E1 region, known for vector adaptation, enhanced transmissibility, and higher epidemic potential (9), and also lacks the internal poly(A) within the 3′UTR otherwise present in the prototypic S-27 strain (10). The phylogenetic tree of the CHIKV whole genome sequences from all lineages built using MEGA11 (v.11.0.13) reveals that CHIKV-IS belongs to the ECSA (East/Central/South African) lineage, sharing nearly 99% sequence identity to several Indian isolates (Fig. 1).

## Data Availability

The complete genome sequence of the chikungunya virus Indian outbreak strain reported here was deposited in GenBank under accession number PP349434. Raw sequence data were deposited in the National Center for Biotechnology Information Sequence Read Archive (SRA) under SRA accession number SRR28383157 and BioProject accession number PRJNA1089339.
